# Editorial Note: Apoptosis by [Pt(*O,O′-*acac)(γ-acac)(DMS)] requires PKC-δ mediated p53 activation in malignant pleural mesothelioma

**DOI:** 10.1371/journal.pone.0353725

**Published:** 2026-07-14

**Authors:** The PLOS One Editors

After this article [1,2] was published, concerns were raised regarding Figs 3–5. Specifically,

- The following panels in Figs 3–5 appear to overlap with one another:
  - The ZL34 β-actin panel of Fig 3A and the ZL55 β-actin panel of Fig 4A, when flipped horizontally.
  - The ZL55 β-actin panel of Fig 3A and the ZL34 β-actin panel of Fig 4A, when flipped horizontally.
  - Lanes 2-4 of the ZL55 Bcl-2 panel of Fig 3A and lanes 2-4 of the ZL34 cyt panel of Fig 5A, when stretched vertically.
  - Lanes 1-2 of the ZL55 BAX panel of Fig 3A and lanes 2-3 of the PKC-α mem panel of Fig 5A, when stretched vertically.
  - Lanes 1-2 of the p-JNK1/2 panels in Figs 5C and 5D.
  - Lane 1 of the Bcl-2 panel in Fig 5C and lane 3 of the Bcl-2 panel in Fig 5D.
- The lower bands of lanes 2 and 5 of the Casp-9 panel of Fig 5C appear similar to each other.
- Similarities were noted between the backgrounds of lanes 2 and 6 in the ZL34 p-p38MAPK panel of Fig 3A.
- The ZL55 p-p38MAPK panel of Fig 4A appears to overlap with the [Pt(*O*,*O′*-acac)(γ-acac)(DMS)] p38MAPK panel in Fig 5A of [3].

The corresponding author provided the original, underlying images for all panels of concern (S1 File). They further stated that the ZL55 p-p38MAPK panel in Fig 4A of [1] was intentionally reused from the [Pt(*O*,*O′*-acac)(γ-acac)(DMS)] p38MAPK panel in Fig 5A of [3], as stated in the Ptac2S-induces MAPKs phosphorylation section of the Results, as the panels originated from the same time-course experiment examining the activation of p38MAPK in ZL55 cells following treatment with the platinum complex.

Following editorial review of the original, underlying images (provided here as S1 File), PLOS considers all the above concerns resolved.

In addition, contrary to the declaration in the Data Availability statement, the original raw data files supporting the article’s results were not provided with the article. The authors have provided the data as S2 File. With this notice, all relevant data are now provided.

## Supporting information

S1 File Original blots underlying all panels of concern within Figs 3–5. (ZIP)

S2 File Original quantitative data underlying all Figs. (PDF)

## References

[1] Muscella A, Vetrugno C, Cossa LG, Antonaci G, Barca A, De Pascali SA, et al. Apoptosis by [Pt(*O*,*O’*-acac)(γ-acac)(DMS)] requires PKC-δ mediated p53 activation in malignant pleural mesothelioma. PLoS One. 2017;12(7):e0181114. doi: 10.1371/journal.pone.0181114 28704484 PMC5507537

[2] Muscella A, Vetrugno C, Cossa LG, Antonaci G, Barca A, De Pascali SA, et al. Correction: Apoptosis by [Pt(*O*,*O’*-acac)(γ-acac)(DMS)] requires PKC-δ mediated p53 activation in malignant pleural mesothelioma. PLoS One. 2018;13(2):e0193030. doi: 10.1371/journal.pone.0193030 29432478 PMC5809079

[3] Muscella A, Vetrugno C, Cossa LG, Antonaci G, De Nuccio F, De Pascali SA, et al. *In Vitro* and *In Vivo* Antitumor Activity of [Pt(*O*,*O’*-acac)(γ-acac)(DMS)] in Malignant Pleural Mesothelioma. PLoS One. 2016;11(11):e0165154. doi: 10.1371/journal.pone.0165154 27806086 PMC5091852

[4] Antonaci G, Cossa LG, Muscella A, Vetrugno C, De Pascali SA, Fanizzi FP, et al. [Pt(*O*,*O’*-acac)(γ-acac)(DMS)] Induces Autophagy in Caki-1 Renal Cancer Cells. Biomolecules. 2019;9(3):92. doi: 10.3390/biom9030092 30845773 PMC6468382

