# Supplementary figures and images for: Editorial Note: Apoptosis by [Pt(O,O′-acac)(γ-acac)(DMS)] requires PKC-δ mediated p53 activation in malignant pleural mesothelioma
Source: PLoS One. 2026 Jul 14;21(7):e0353725. doi: 10.1371/journal.pone.0353725 (PMC13367687; doi:10.1371/journal.pone.0353725)

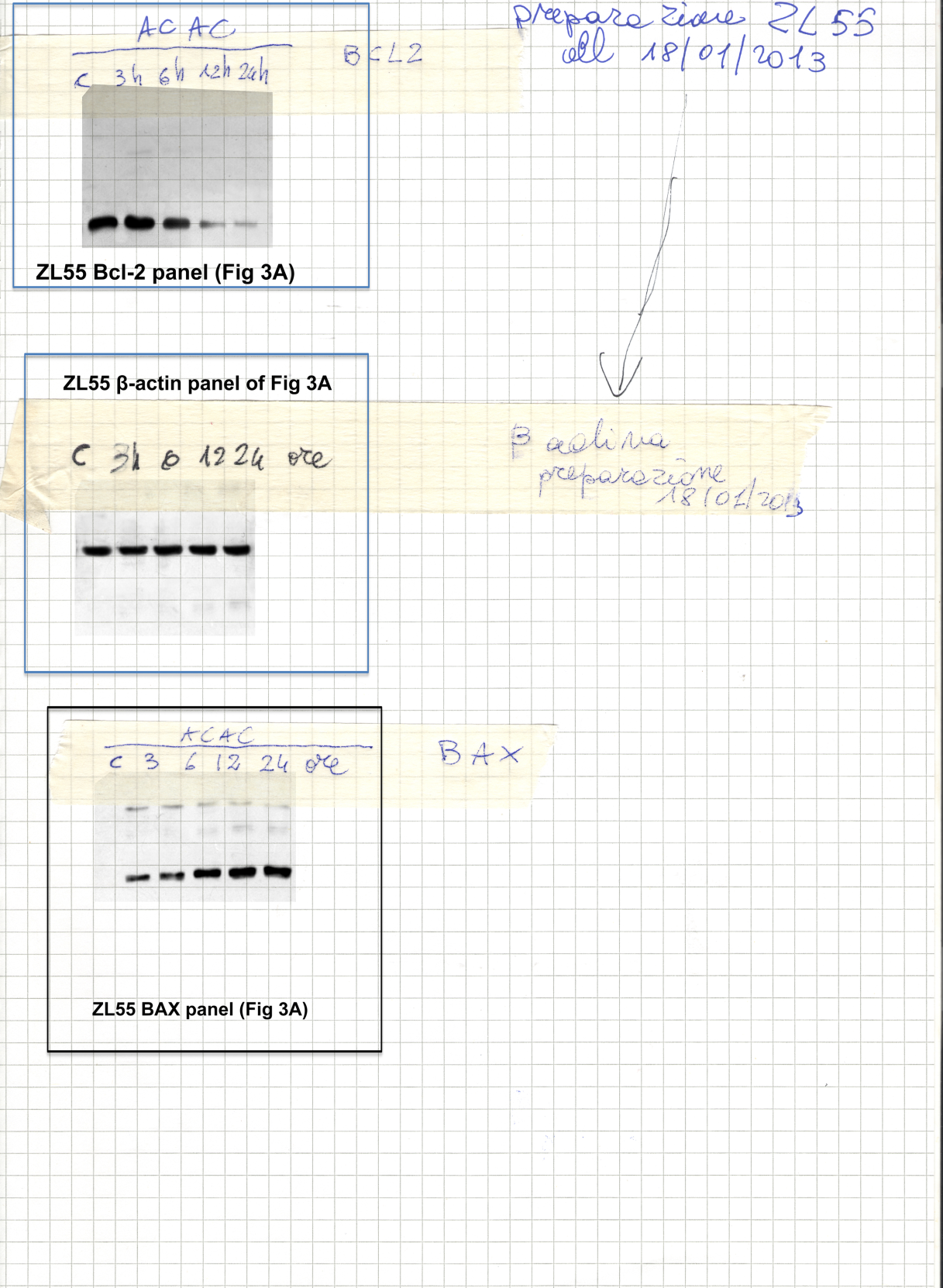

Supplement: S1 File — (ZIP) [file pone.0353725.s001.zip › S1 - Original blots underlying panels within Figs 2-5/S1A.tif]

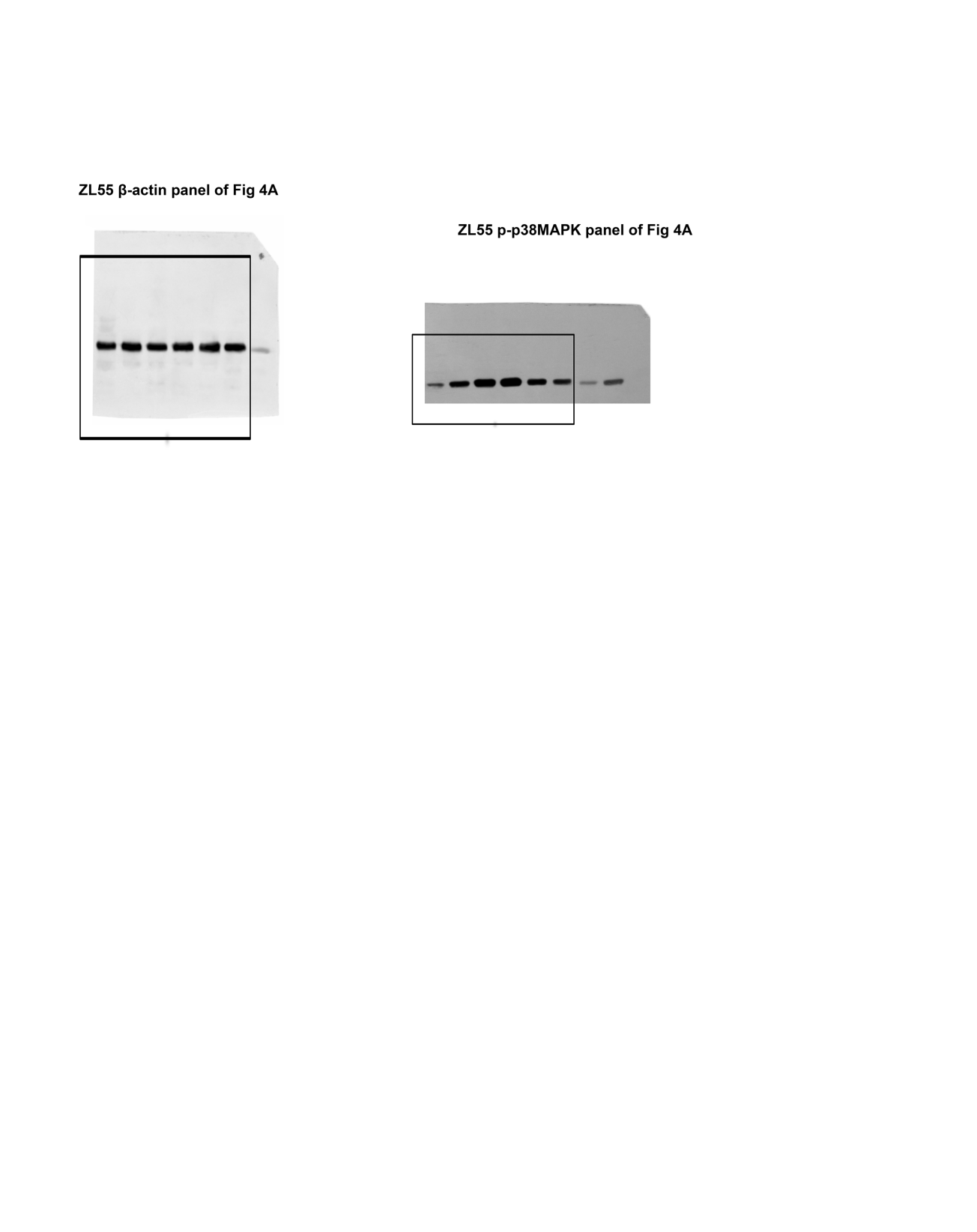

Supplement: S1 File — (ZIP) [file pone.0353725.s001.zip › S1 - Original blots underlying panels within Figs 2-5/S1B.tif]

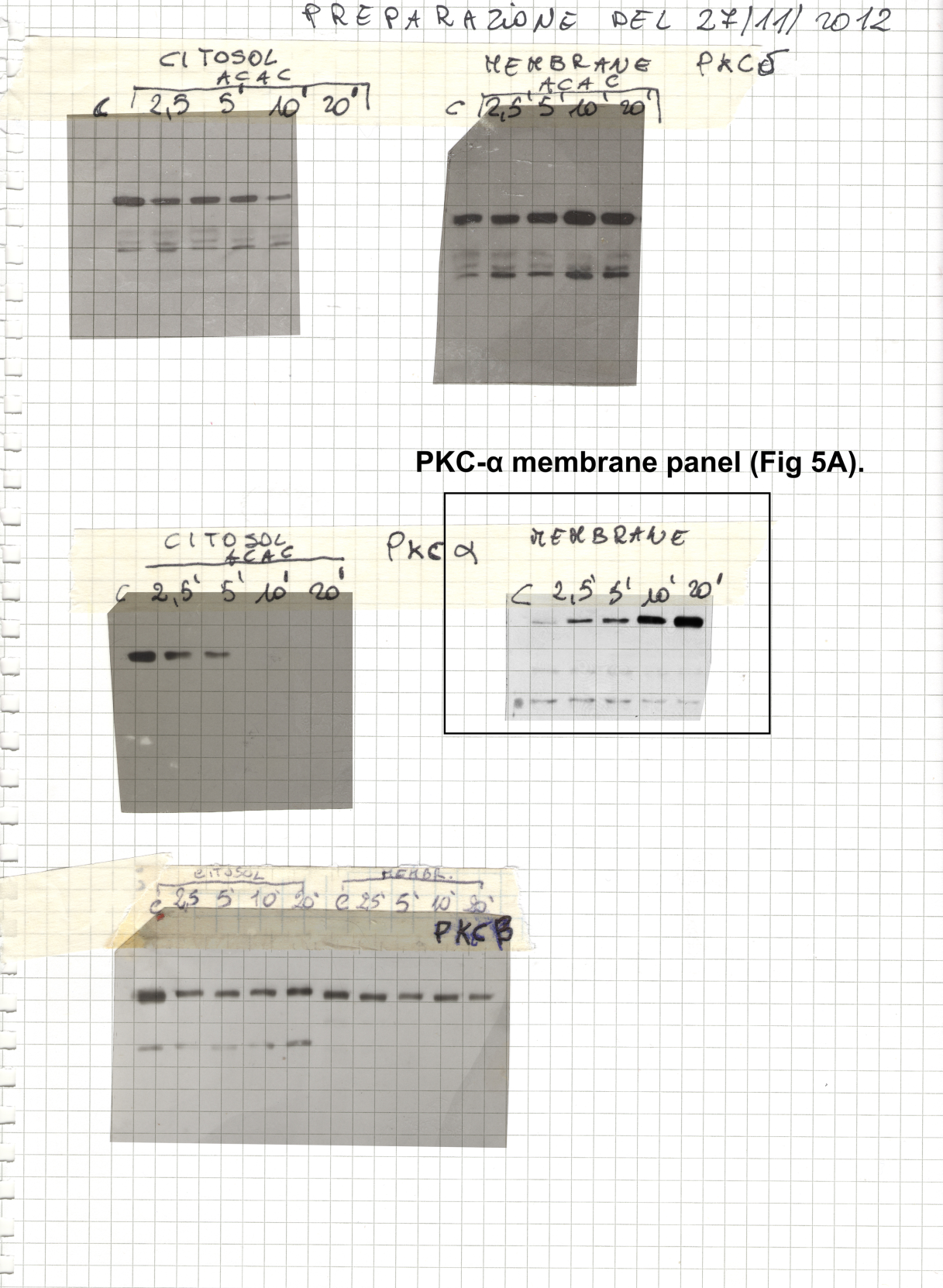

Supplement: S1 File — (ZIP) [file pone.0353725.s001.zip › S1 - Original blots underlying panels within Figs 2-5/S1C.tif]

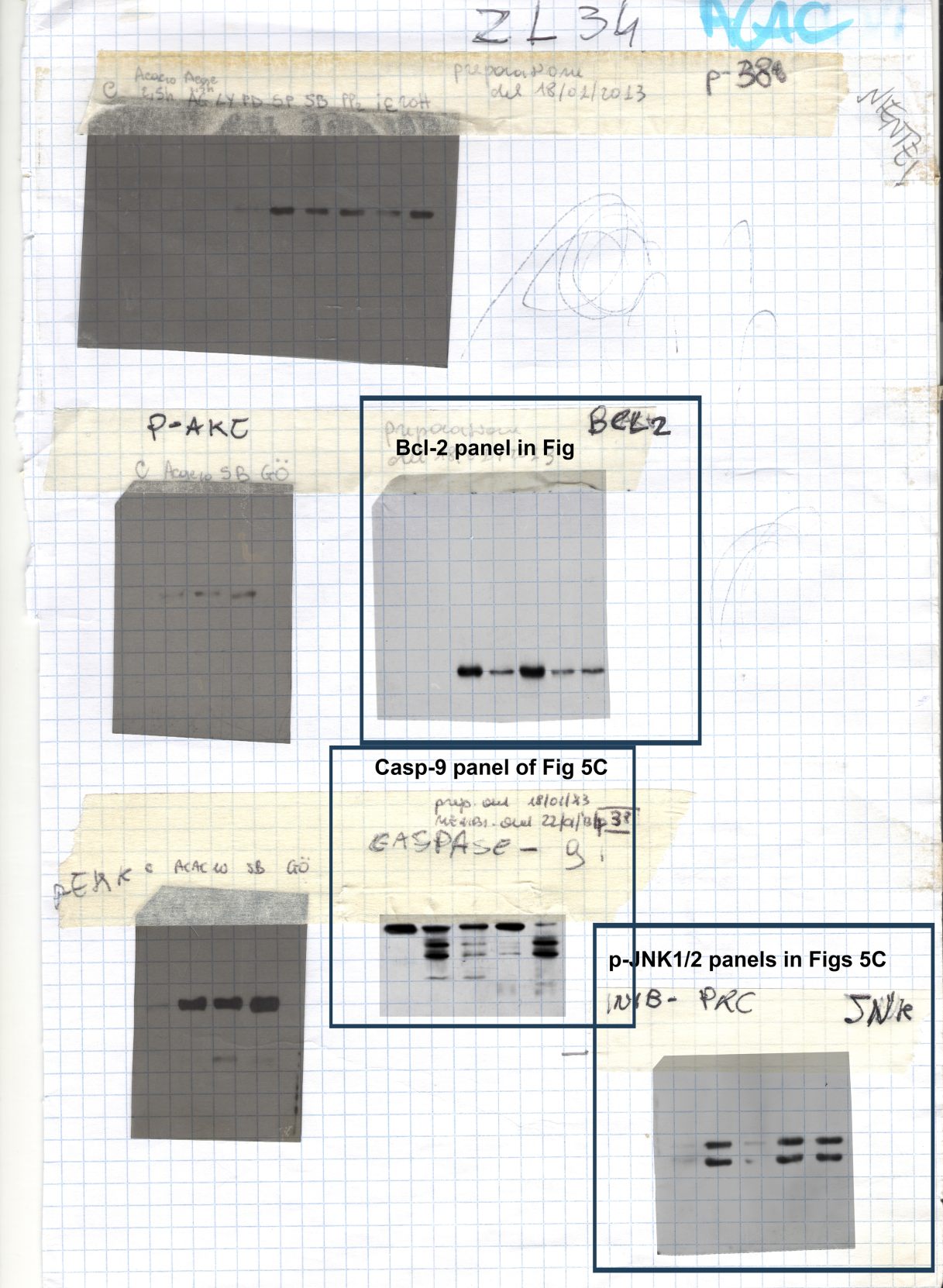

Supplement: S1 File — (ZIP) [file pone.0353725.s001.zip › S1 - Original blots underlying panels within Figs 2-5/S1D.tif]

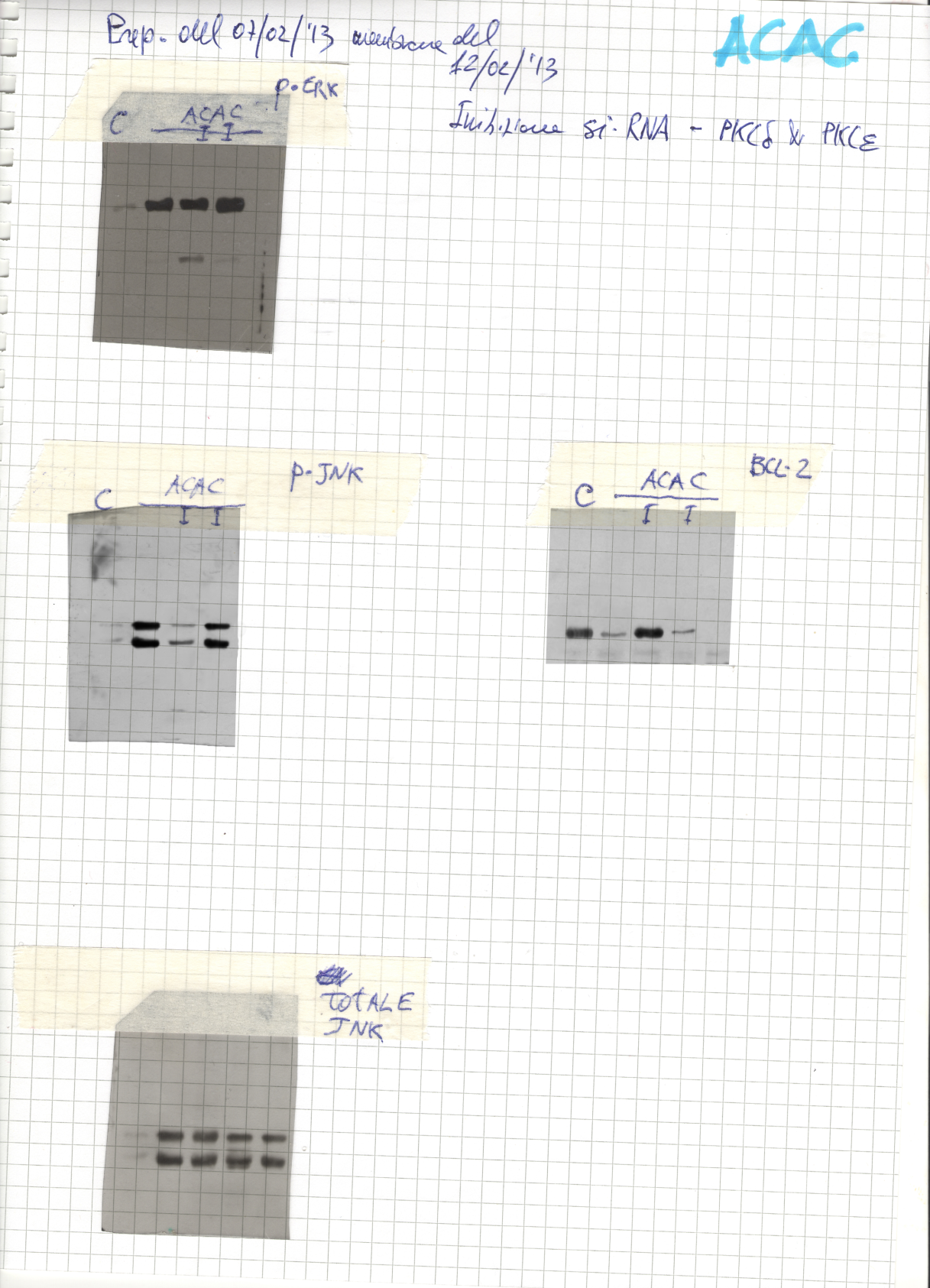

Supplement: S1 File — (ZIP) [file pone.0353725.s001.zip › S1 - Original blots underlying panels within Figs 2-5/S1E.tif]

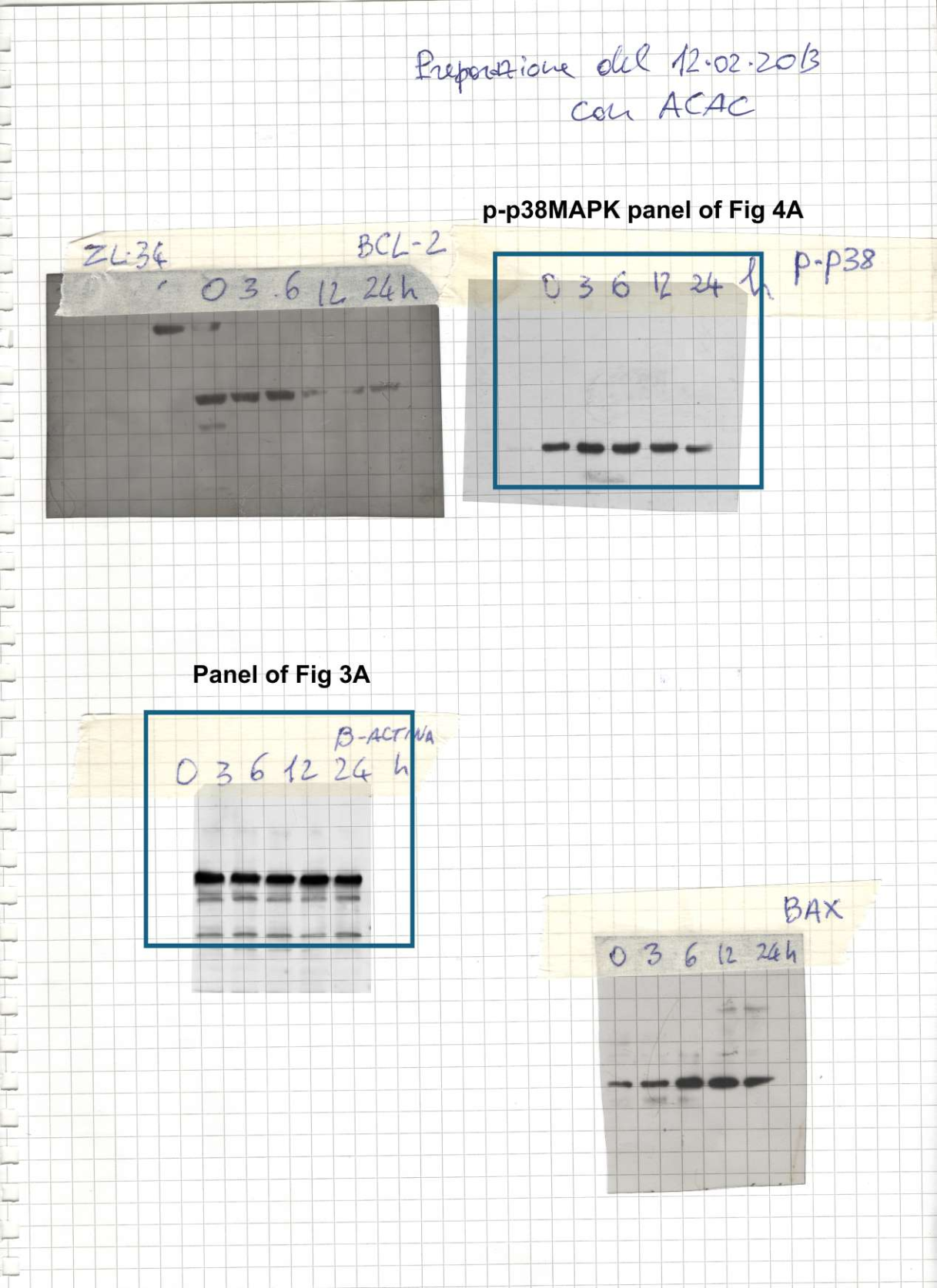

Supplement: S1 File — (ZIP) [file pone.0353725.s001.zip › S1 - Original blots underlying panels within Figs 2-5/S1F.tif]

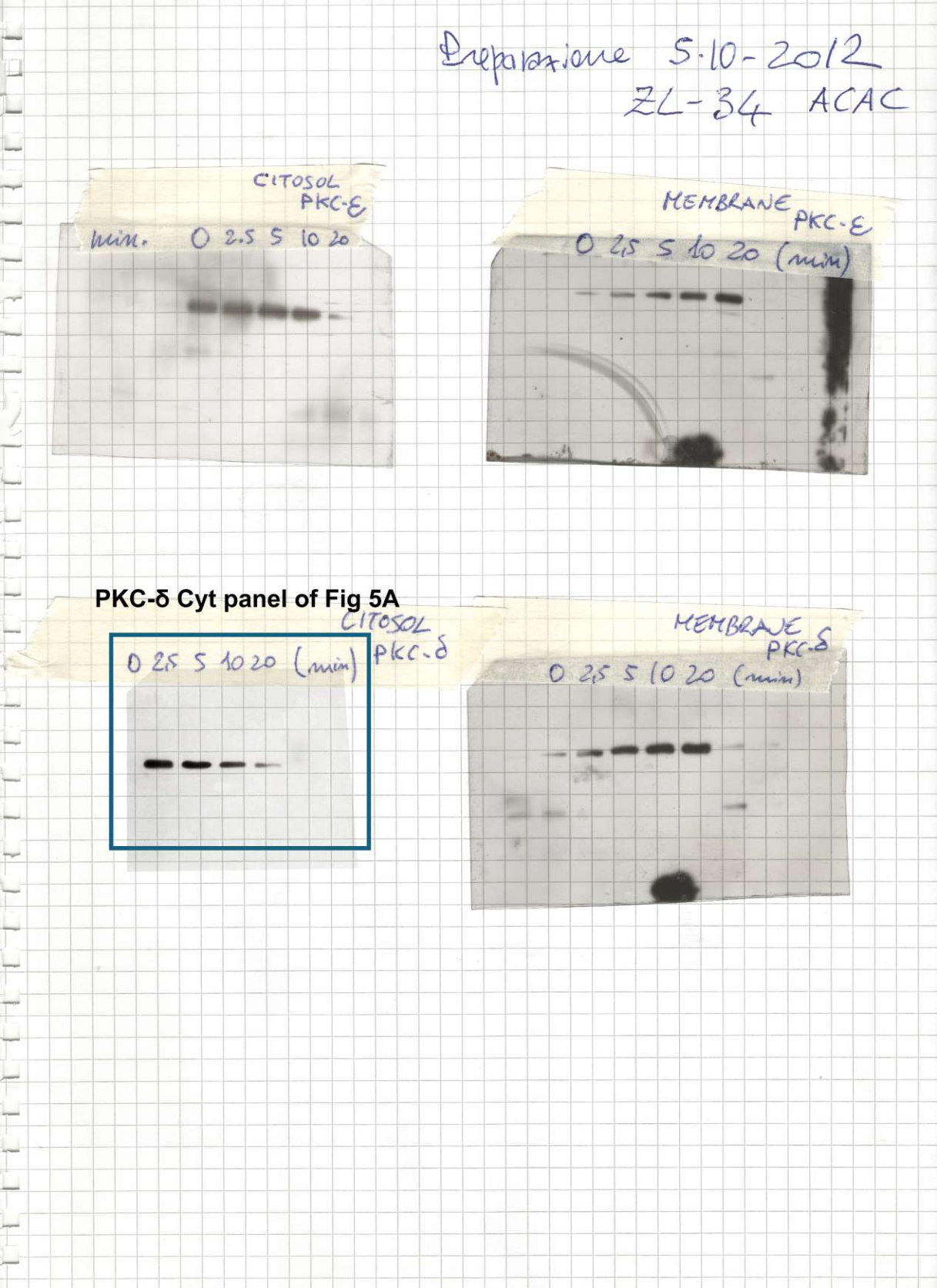

Supplement: S1 File — (ZIP) [file pone.0353725.s001.zip › S1 - Original blots underlying panels within Figs 2-5/S1G.tif]
