# Supplementary material for: Editorial Note: Apoptosis by [Pt(O,O′-acac)(γ-acac)(DMS)] requires PKC-δ mediated p53 activation in malignant pleural mesothelioma
Source: PLoS One. 2026 Jul 14;21(7):e0353725. doi: 10.1371/journal.pone.0353725 (PMC13367687; doi:10.1371/journal.pone.0353725)
Supplement: S2 File — (PDF) [file pone.0353725.s002.pdf]

Controllo

Animale	Settimana 0	Settimana 1	Settimana 2	Settimana 3	Settimana 4	Settimana 5
Topo 1	42,1	85,4	165,2	212,4	248,5	281,4
Topo 2	39,5	72,1	134,1	188,5	215,3	231,45
Topo 3	45	94,3	185,4	234,1	278,4	262,1
Topo 4	38	68,5	122	172,3	202,1	274,8
Topo 5	41,2	82	158,3	208	244	291,5
Topo 6	46,4	99,1	192,1	245,6	292,6	295,6
Topo 7	37,8	74,2	141,5	181,2	218,4	312
Topo 8	44	88,4	173,4	222,1	260,7	341,2
Media	41,75	83	159	208,025	245	286,25625
SD	3,216031272	10,88458937	24,88292587	25,87363523	31,78301433	32,81645316

Cisplatin 10 mg/Kg

Animale	Settimana 0	Settimana 1	Settimana 2	Settimana 3	Settimana 4	Settimana 5
Topo 1	41,5	82,1	144,2	174,3	210,5	281,4
Topo 2	43	78,4	128,5	155,1	184,2	175,4
Topo 3	39	88	156,1	191	228,4	212,8
Topo 4	44,2	71,3	119,4	144,2	171,3	244,1
Topo 5	38,5	76,5	135	168,4	202,1	256,3
Topo 6	42	84,2	149,3	182,5	219	261,9
Topo 7	40,1	79	131,2	161	196,4	281,4
Topo 8	45,3	91,1	162,5	202,1	242,5	301,6
Media	41,7	81,325	140,775	172,325	206,8	251,8625
SD	2,423692578	6,399944196	14,75434561	19,17630606	23,31412815	41,00842901

Ptac2S 10 mg/Kg

Animale	Settimana 0	Settimana 1	Settimana 2	Settimana 3	Settimana 4	Settimana 5
Topo 1	40,2	48,5	61,2	84,1	112,4	152,3
Topo 2	42,5	52,1	68,4	92,5	124,1	74,2
Topo 3	38,4	44,3	54	73,2	95,3	101,55
Topo 4	43,1	56	74,1	101,4	132,5	122,4
Topo 5	39	46,2	58,5	81	106	136,1
Topo 6	41,6	51	66,2	94,3	121,2	144,8
Topo 7	40,5	47,4	60,1	82,5	108,4	153
Topo 8	44,3	54,1	71,5	96,2	126,5	185,4
Media	41,2	49,95	64,25	88,15	115,8	133,71875
SD	2,043805973	4,038033468	6,920569754	9,411694853	12,3868594	34,27174948

Gruppo di Trattamento	Media Dimensione Tumor	% del Controllo	Significatività vs Saline
Saline (Controllo)	285,11	100	-
Cisplatin 10 mg/Kg	251,87	88,3	n.s.
Ptac2S 10 mg/Kg	133,72	46,9	*** (p<0.001)

ANALISi

Origine Variazione	Gradi di Libertà (Df)	F-Value	p-value	Significatività
Tra i Gruppi (Trattamenti)	2	25,41	0,0001	Estremamente Significativo (***)
Entro Gruppi (Errore)	21			

POST-HOC

Confronto Coppia	Differenza Medie (mm³)	95% CI Inf	95% CI Sup	p-value	Esito
Cisplatin vs Saline	-33,24	-86,5	20,02	0,2831	Non Significativo (n.s.)
Ptac2S vs Saline	-151,39	-204,65	-98,13	0,0001	Altamente Significativo (***)
Ptac2S vs Cisplatin	-118,15	-171,41	-64,89	0,0001	Altamente Significativo (***)

24 ore di trattamento

Concentrazione (µM)	Composto	Exp 1	Exp 2	Exp 3	Exp 4	Exp 5	Exp 6	MEDIA		Bonferroni
0	Cisplatin	100	100	100	100	100	100	100		a
1	Cisplatin	103,539767	92,65976005	93,61064	99,47979	97,20537	103,9047	98,4	4,8	a
10	Cisplatin	89,8398772	96,4540371	83,7172	99,12648	89,27207	92,39033	91,8	5,5	a
100	Cisplatin	63,6861142	53,65741959	56,57781	63,63061	66,13534	63,51271	61,2	4,9	c
200	Cisplatin	61,4336534	49,05201939	61,31884	57,1508	55,43555	63,00913	57,9	5,2	c
0	Ptac2S	100	100	100	100	100	100	100	0	a
1	Ptac2S	74,7305229	72,34907542	85,94013	77,95242	79,33754	72,29031	77,1	5,2	b
10	Ptac2S	52,0185958	61,92368962	54,3928	50,17801	57,76735	49,51955	54,3	4,8	c
100	Ptac2S	34,9149447	33,97843605	28,03274	31,86389	33,17282	40,83718	33,8	4,2	d
200	Ptac2S	33,3089585	32,7336574	22,5193	25,57701	29,27204	32,98903	29,4	4,5	d

48 ore di trattamento

Concentrazione (µM)	Composto	Exp 1	Exp 2	Exp 3	Exp 4	Exp 5	Exp 6	MEDIA	DEV.ST.	Bonferroni
0	Cisplatin	100	100	100	100	100	100	100	0	a
1	Cisplatin	89,7595246	95,51786408	80,46283	92,84555	90,20161	88,81262	89,6	5,1	a
10	Cisplatin	63,5391093	66,10997284	77,98767	73,07074	65,87527	74,61724	70,2	5,8	b
100	Cisplatin	35,5566163	35,29681046	33,40047	31,62447	22,87573	30,24591	31,5	4,7	c
200	Cisplatin	30,2590087	29,09278071	22,61408	29,26116	29,56587	38,00711	29,8	4,9	c
0	Ptac2S	100	100	100	100	100	100	100	0	a
1	Ptac2S	69,0049004	57,86414705	67,33629	70,42662	76,43161	65,73644	67,8	6,1	b
10	Ptac2S	34,5644226	40,3649529	29,24598	31,73766	31,30875	24,17823	31,9	5,4	c
100	Ptac2S	10,0970124	7,178246906	11,2493	14,83985	24,25316	13,38243	13,5	5,9	d
200	Ptac2S	9,14218058	12,26193698	10,13797	2,975304	17,57583	15,10678	11,2	5,1	d

Valori di IC50 calcolati (µmol L⁻¹)

Linea Cellulare	Cisplatin (µM)	Ptac2S (µM)	
**ZL34**	48.63 ± 0.72	4.64 ± 0.13	10.5 volte più potente

One-Way ANOVA e Post-Hoc

ANOVA: Cisplatin 24h

Valore F	p-value	Significatività
48,12	< 0.0001	mente Significativo (***)

ANOVA: Ptac2S 24h

Valore F	p-value	Significatività
124,53	< 0.0001	mente Significativo (***)

ANOVA: Cisplatin 48h

Valore F	p-value	Significatività
92,45	< 0.0001	mente Significativo (***)

ANOVA: Ptac2S 48h

Valore F	p-value	Significatività
218,19	< 0.0001	mente Significativo (***)

Saggio di Clonogenica (Crescita 15 giorni)

Concentrazione (µM)	Composto	Exp 1	Exp 2	Exp 3	Exp 4	Exp 5	Exp 6	MEDIA	DEV.ST.	Bonferroni
0	Cisplatin	100	100	100	100	100	100	100	0	a
0,5	Cisplatin	94,11323343	97,79538332	103,0824797	96,60921442	106,0329158	96,96677333	99,1	4,5	a
1	Cisplatin	89,9998931	96,71372737	99,2236423	97,38239867	88,24993068	100,8304079	95,4	5,1	a
2,5	Cisplatin	77,55036124	69,00776386	77,90119505	75,38108212	68,78750258	76,57209514	74,2	4,2	b
5	Cisplatin	72,04968694	71,70989049	75,08853096	61,89417247	69,01631035	67,24140879	69,5	4,6	b
0	Ptac2S	100	100	100	100	100	100	100	0	a
0,5	Ptac2S	47,08183161	47,50042939	57,80127764	54,40165405	51,69502037	58,31978694	52,8	4,9	b
1	Ptac2S	39,18596576	43,37824653	44,26941927	35,75081611	43,06776224	33,74779008	39,9	4,4	c
2,5	Ptac2S	24,81577319	33,01326659	27,07376501	33,58630556	28,71778022	23,79310943	28,5	4,1	d
5	Ptac2S	8,748851684	8,382944292	9,666764501	13,16911813	4,595700841	14,23662056	9,8	3,5	d

Saggio Clonogenicità - Cisplatin 15d (P < 0.001)

Origine della Variazion	Gradi di Lil	Valore F	p-value	Significatività
Tra i Gruppi (Trattamer	4	18,74	< 0.001	Significativo (***)
Entro i Gruppi (Residuc	25			

Saggio Clonogenicità - Ptac2S 15d (P < 0.0001)

Origine della Variazion	Gradi di Lil	Valore F	p-value	Significatività
Tra i Gruppi (Trattamer	4	164,88	< 0.0001	Significativo (***)

Real-Time PCR Quantification mRNA Expression Levels

p53 mRNA (ZL34)

Time (min)	Exp 1	Exp 2	Exp 3	Exp 4	Exp 5	Exp 6	MEDIA	DEV.ST.
0	1	1	1	1	1	1	1	
15	2,080212892	1,793524682	2,30191714	2,205035912	2,641394461	2,297914914	2,22	0,28
30	3,208138016	2,991978039	3,615297456	3,352655753	3,421374596	3,270556139	3,31	0,21
45	3,200774652	3,9904648	3,874986429	3,804085052	3,924149846	3,405539221	3,7	0,32
60	3,688127598	3,364740311	3,460647936	3,515273655	3,024574957	3,646635543	3,45	0,24
PFT-α + Ptac2S	1,071246567	0,647836276	0,970526535	1,716082129	1,000074714	0,71423378	1,02	0,38

Pannello B - p53 mRNA (ZL55)

Time (min) / Condizione	Exp 1	Exp 2	Exp 3	Exp 4	Exp 5	Exp 6	MEDIA	DEV.ST.
0	1	1	1	1	1	1	1	0
15	1,802689035	1,963222908	1,669814358	1,674900278	2,195356977	2,094016445	1,9	0,22
30	3,56894912	3,476333007	3,792135152	2,942158965	3,562712674	3,777711083	3,52	0,31
45	5,859281536	4,997040589	5,684198597	5,92322789	5,098028293	5,438223096	5,5	0,39
60	3,1667372	3,026174727	2,918865163	2,741822468	2,330223063	3,276177379	2,91	0,34
PFT-α + Ptac2S	0,570410093	1,173971571	1,238628978	0,927799011	1,182448783	1,206741563	1,05	0,26

Pannello C - BAX mRNA (ZL34)

Time (min) / Condizione	Exp 1	Exp 2	Exp 3	Exp 4	Exp 5	Exp 6	MEDIA	DEV.ST.
0	1	1	1	1	1	1	1	0
15	1,868550835	1,262146445	1,677395694	1,600165156	1,0724581	1,21928377	1,45	0,31
30	2,431831883	1,939537945	2,349616811	2,202310217	2,066164621	2,210538524	2,2	0,18
45	2,669555466	2,396698462	2,509254435	3,025851013	2,156880624	2,541760002	2,55	0,29
60	2,478974909	2,630323095	2,498254822	2,707219197	2,806080006	2,17914797	2,55	0,22
PFT-α + Ptac2S	1,157089823	1,102168752	0,59762632	1,413193826	0,857650565	0,932270714	1,01	0,28

Pannello C - BAX mRNA (ZL55)

Time (min) / Condizione	Exp 1	Exp 2	Exp 3	Exp 4	Exp 5	Exp 6	MEDIA	DEV.ST.
0	1	1	1	1	1	1	1	0
15	2,028520043	1,45790184	1,34048482	1,531335204	1,654112818	1,707645275	1,62	0,24
30	3,095284945	3,408840645	2,628287695	2,490479249	2,972505951	2,864601514	2,91	0,33
45	3,189240456	2,933422881	3,336458492	3,332968496	3,146977224	3,74093245	3,28	0,27
60	2,643876221	3,29939326	2,824392921	2,74175917	2,648263136	3,302315292	2,91	0,31
PFT-α + Ptac2S	0,744737939	0,796080007	0,829526334	1,175854415	1,487663388	1,146137918	1,03	0,29

Pannello D - Bcl-2 mRNA (ZL34)

Time (min) / Condizione	Exp 1	Exp 2	Exp 3	Exp 4	Exp 5	Exp 6	MEDIA	DEV.ST.
0	1	1	1	1	1	1	1	0
15	0,698755326	0,709558436	0,520873237	0,601549548	0,662102937	0,647160515	0,64	0,07
30	0,540283829	0,646137766	0,5155783	0,517245507	0,559353902	0,521400695	0,55	0,05
45	0,102829483	0,238638246	0,170417679	0,231561237	0,245997372	0,090555983	0,18	0,07
60	0,099203271	0,03875375	0,082268267	0,103535984	0,025618286	0,010620444	0,06	0,04
PFT-α + Ptac2S	0,924899765	0,874969487	0,843245868	0,976726088	0,899931319	1,000227473	0,92	0,06

Pannello D - Bcl-2 mRNA (ZL55)

Time (min) / Condizione	Exp 1	Exp 2	Exp 3	Exp 4	Exp 5	Exp 6	MEDIA	DEV.ST.
0	1	1	1	1	1	1	1	0
15	0,712087698	0,557615012	0,576305681	0,672889562	0,664594473	0,656507573	0,64	0,06
30	0,578310425	0,58185294	0,408385051	0,50098194	0,485923893	0,444545752	0,5	0,07
45	0,07569758	0,042246136	0,1467439	0,136760583	0,173598716	0,144953086	0,12	0,05
60	0,08768393	0,098658848	0,04339302	0,047710349	0,104124514	0,038429339	0,07	0,03
PFT-α + Ptac2S	0,990303938	1,004604086	0,908420465	0,926346358	0,93821439	0,872110762	0,94	0,05

ANOVA: p53 mRNA in ZL34 Cells

Origine della Variazione	Gradi di Libertà (Df)	Valore F	p-value	Significatività
Tra i Gruppi (Trattamenti/Tempo)	5	38,64	<0.0001	Altamente Significativo (***)
Entro i Gruppi (Residuo/Errore)	30			

ANOVA: p53 mRNA in ZL55 Cells

Origine della Variazione	Gradi di Libertà (Df)	Valore F	p-value	Significatività
Tra i Gruppi (Trattamenti/Tempo)	5	54,19	<0.0001	Altamente Significativo (***)
Entro i Gruppi (Residuo/Errore)	30			

ANOVA: BAX mRNA in ZL34 Cells

Origine della Variazione	Gradi di Libertà (Df)	Valore F	p-value	Significatività
Tra i Gruppi (Trattamenti/Tempo)	5	22,41	<0.0001	Altamente Significativo (***)
Entro i Gruppi (Residuo/Errore)	30			

ANOVA: BAX mRNA in ZL55 Cells

Origine della Variazione	Gradi di Libertà (Df)	Valore F	p-value	Significatività
Tra i Gruppi (Trattamenti/Tempo)	5	41,85	<0.0001	Altamente Significativo (***)
Entro i Gruppi (Residuo/Errore)	30			

Vitalità Cellulare e Silenziamento delle PKC

Cellule ZL34 - 24 ore di incubazione

Concentrazione (μM)	Cisplatin E1	Cisplatin E2	Cisplatin E3	Cisplatin E4	Cisplatin E5	MEDIA CIS	DEV.ST CIS	Ptac2S E1	Ptac2S E2	Ptac2S E3	Ptac2S E4	Ptac2S E5	MEDIA	DEV.ST
0 μM (Controllo)	100	100	100	100	100	100	#NOME?	100	100	100	100	100	100	0
1 μM	107,5766802	96,74835253	98,20606116	96,10082025	96,86808585	99,1	#NOME?	77,01871068	74,63805894	81,18464975	69,36991198	81,78866866	76,8	5,1
10 μM	98,77283924	89,11228521	95,8334146	87,64419394	86,13726702	91,5	#NOME?	51,83873435	48,19821991	56,71506822	59,70274305	50,54523447	53,4	4,7
100 μM	62,79467383	67,20130123	56,43129991	58,16399441	61,40873062	61,2	#NOME?	34,97585266	32,00861529	32,24211135	40,51135313	30,76206757	34,1	3,9
200 μM	64,3493498	62,16395085	56,31943021	54,50287064	53,16439851	58,1	#NOME?	27,7185565	25,14357007	33,78441263	26,6628144	34,19064639	29,5	4,2

Cellule ZL34 - 48 ore di incubazione

Concentrazione (μM)	Cisplatin E1	Cisplatin E2	Cisplatin E3	Cisplatin E4	Cisplatin E5	MEDIA CIS	DEV.ST CIS	Ptac2S E1	Ptac2S E2	Ptac2S E3	Ptac2S E4	Ptac2S E5	MEDIA	DEV.ST
0 μM (Controllo)	100	100	100	100	100	100	#NOME?	100	100	100	100	100	100	0
1 μM	86,0907219	91,67418594	85,41801535	90,75257361	95,5645032	89,9	#NOME?	64,15021586	64,83370341	68,55899883	76,78706668	61,67001521	67,2	5,9
10 μM	65,65940526	69,63440373	74,43120348	68,34121869	78,43376885	71,3	#NOME?	34,60816101	26,11819164	38,255625	25,2131612	32,80486116	31,4	5,6
100 μM	30,44206923	38,79779291	26,68959994	29,67590621	31,89463172	31,5	#NOME?	17,18362919	12,78359665	20,91732066	8,764271813	9,851181694	13,9	5,1
200 μM	30,14243813	26,39481216	23,74664032	35,41931417	33,29679521	29,8	#NOME?	2,394950094	14,22068007	15,09972069	5,242368873	15,54228027	10,5	6,2

Silenziamento Isoforme PKC tramite siRNA (Cellule MPM trattate con Ptac2S 5 μM)

Condizione Sperimentale (siRNA)	Exp 1	Exp 2	Exp 3	Exp 4	Exp 5	MEDIA	DEV.ST.	Post-hoc Dunn
Controllo (siRNA / No Ptac2S)	100	100	100	100	100	100	#NOME?	a
Ptac2S (5 μM) + siRNA Scramble	49,80639545	57,31158665	59,86796446	64,86678143	64,14727201	59,2	#NOME?	b
Ptac2S (5 μM) + siRNA PKC-δ	90,20083026	82,56457617	99,56629486	93,35571683	84,81258188	90,1	#NOME?	c
Ptac2S (5 μM) + siRNA PKC-ε	66,1453186	80,35723777	74,85109809	63,724889	67,42145654	70,5	#NOME?	d
Ptac2S (5 μM) + siRNA PKC-α	49,76236053	36,95692376	41,67525379	48,45017241	40,15528951	43,4	#NOME?	e

ANOVA: Cisplatin Dose-Response Curve ZL34 (24h)

Origine della Variazione	Gradi di Libertà (Df)	Valore F	p-value	Significatività
Tra i Gruppi (Trattamenti)	4	48,12	< 0.0001	Estremamente Significativo (***)
Entro i Gruppi (Residuo/Errore)	20			

ANOVA: Ptac2S Dose-Response Curve ZL34 (24h)

Origine della Variazione	Gradi di Libertà (Df)	Valore F	p-value	Significatività
Tra i Gruppi (Trattamenti)	4	114,65	< 0.0001	Estremamente Significativo (***)
Entro i Gruppi (Residuo/Errore)	20			

ANOVA: Cisplatin Dose-Response Curve ZL34 (48h)

Origine della Variazione	Gradi di Libertà (Df)	Valore F	p-value	Significatività
Tra i Gruppi (Trattamenti)	4	93,42	< 0.0001	Estremamente Significativo (***)
Entro i Gruppi (Residuo/Errore)	20			

ANOVA: Ptac2S Dose-Response Curve ZL34 (48h)

Origine della Variazione	Gradi di Libertà (Df)	Valore F	p-value	Significatività
Tra i Gruppi (Trattamenti)	4	187,31	< 0.0001	Estremamente Significativo (***)
Entro i Gruppi (Residuo/Errore)	20			

ANOVA: siRNA PKC Transfection (24h)

Origine della Variazione	Gradi di Libertà (Df)	Valore F	p-value	Significatività
Tra i Gruppi (Trattamenti)	4	64,89	< 0.0001	Estremamente Significativo (***)
Entro i Gruppi (Residuo/Errore)	20			

Knockdown delle PKC in Cellule ZL55

Saggio di Silenziamento siRNA-PKC in Cellule ZL55 (24 ore)

Braccio Sperimentale / Condizione siRNA	Exp 1	Exp 2	Exp 3	Exp 4	Exp 5	MEDIA	DEV.ST.	Bonferroni/Dunn
Controllo (siRNA / No Ptac2S)	100	100	100	100	100	100	0	a
Ptac2S (5 μM) + siRNA Scramble	46,16073723	44,60646547	43,81695417	58,34593039	48,06991274	48,2	5,9	b
Ptac2S (5 μM) + siRNA PKC-δ	84,70395708	77,17907982	79,46234307	67,18997881	75,46464121	76,8	6,4	c
Ptac2S (5 μM) + siRNA PKC-ε	91,33270782	82,62684591	94,76659317	79,88231549	86,89153761	87,1	6,1	c

Report Analisi della Varianza: One-Way ANOVA Cellule ZL55

ANOVA: siRNA PKC-δ/ε Transfection in ZL55 Cells

Origine della Variazione	Gradi di Libertà (Df)	Valore F	p-value	Significatività
Tra i Gruppi (Trattamenti siRNA)	3	72,54	< 0.0001	Estremamente Significativo (***)
Entro i Gruppi (Residuo/Errore)	16			

ZL55 (24h)

	Ptac2S	SB203580 (μM)	SP600125 (μM)	PD98059 (μM)	Exp 1	Exp 2	Exp 3	Exp 4	Exp 5	Media	SD
Controllo	No	-	-	-	100,0	100,0	100,0	100,0	100,0	100,0	0,0
Ptac2S 10 μM	Sì	-	-	-	42,1	54,3	44,8	52,1	47,7	48,2	5,0
Ptac2S + SB203580 1 μM	Sì	1	-	-	65,2	74,1	78,9	66,8	72,0	71,4	5,6
Ptac2S + SB203580 10 μM	Sì	10	-	-	75,2	91,4	85,3	76,1	90,0	83,6	7,6
Ptac2S + SP600125 1 μM	Sì	-	1	-	80,1	95,3	84,1	82,2	92,8	86,9	6,7
Ptac2S + SP600125 10 μM	Sì	-	10	-	78,2	92,4	86,1	80,2	88,6	85,1	5,9
Ptac2S + PD98059 10 μM	Sì	-	-	10	44,1	52,3	46,2	53,1	45,8	48,3	4,1
Ptac2S + PD98059 20 μM	Sì	-	-	20	39,2	54,8	41,1	52,3	45,1	46,5	6,8
**Media**					**65,5**	**76,8**	**70,8**	**70,4**	**72,8**	**71,3**	4,1
**SD**					**21,9**	**20,5**	**23,0**	**17,4**	**23,3**	**21,0**	2,4

ANALISI

	f	F-Value	p-value	Significatività
Tra i Gruppi	7	84,15	< 0.0001	Estremamente Significativo (***)
Entro Gruppi	32			

ZL34 (24h)

	Ptac2S	SB203580 (μM)	SP600125 (μM)	PD98059 (μM)	Exp 1	Exp 2	Exp 3	Exp 4	Exp 5	Media	SD
Controllo	No	-	-	-	100,0	100,0	100,0	100,0	100,0	100,0	0,0
Ptac2S 10 μM	Sì	-	-	-	57,1	62,5	71,3	58,9	69,2	63,8	6,2
Ptac2S + SB203580 1 μM	Sì	1	-	-	66,2	76,4	65,1	73,8	69,0	70,1	4,9
Ptac2S + SB203580 10 μM	Sì	10	-	-	85,5	71,2	88,4	76,9	81,5	80,7	6,8
Ptac2S + SP600125 1 μM	Sì	-	1	-	79,1	91,4	78,2	86,8	91,0	85,3	6,3
Ptac2S + SP600125 10 μM	Sì	-	10	-	89,3	75,4	86,1	79,9	88,8	83,9	6,0
Ptac2S + PD98059 10 μM	Sì	-	-	10	44,2	55,1	46,8	57,9	48,0	50,4	5,8
Ptac2S + PD98059 20 μM	Sì	-	-	20	32,1	48,5	51,4	35,2	46,8	42,8	8,6
**Media**					**69,2**	**72,6**	**73,4**	**71,2**	**74,3**	**72,1**	2,0
**SD**					**23,5**	**17,4**	**18,4**	**20,0**	**19,7**	**19,2**	2,3

	Gradi di Libertà (Df)	F-Value	p-value	Significatività
Tra i Gruppi	7	65,42	< 0.0001	Estremamente Significativo (***)
Entro Gruppi	32			
